# The association between diets and periodontitis: a bidirectional two-sample Mendelian randomization study

**DOI:** 10.3389/fgene.2024.1398101

**Published:** 2024-05-31

**Authors:** Xiaoyu Yang, Jingchan Wang, Houlin Hong, Xing Feng, Xiumei Zhang, Jinlin Song

**Affiliations:** ^1^ College of Stomatology, Chongqing Medical University, Chongqing, China; ^2^ Chongqing Key Laboratory of Oral Diseases and Biomedical Sciences, Chongqing Medical University, Chongqing, China; ^3^ Chongqing Municipal Key Laboratory of Oral Biomedical Engineering of Higher Education, Chongqing Medical University, Chongqing, China; ^4^ Department of Community Health and Social Sciences, Graduate School of Public Health and Health Policy, City University of New York, New York City, NY, United States; ^5^ The First Affiliated Hospital of Anhui Medical University, Hefei, China

**Keywords:** periodontitis, dietary habits, dietary antioxidants, mendelian randomization, oral health

## Abstract

**Background:**

Periodontitis, a complex inflammatory condition, has been associated with dietary habits and antioxidants. While the association between certain dietary patterns and periodontitis has been documented, the bidirectional relationship remains unclear. This study utilizes Mendelian randomization (MR) analysis to investigate the bidirectional associations between dietary factors comprising dietary antioxidants, and periodontitis.

**Methods:**

Employing a two-sample MR approach, this study analyzed genome-wide association study (GWAS) data on diets and periodontitis from large databases and published literature. Instrumental variables (IVs) were selected and filtered based on genetic variants associated with dietary factors and periodontitis, respectively. Various MR methods, including Inverse Variance Weighted, MR-Egger, Weighted Median, Weighted Mode, and Simple Mode were applied to assess the bidirectional associations. Sensitivity analyses were conducted to validate the robustness of the findings.

**Results:**

Our analysis revealed significant associations between certain dietary factors and the risk of periodontitis. Specifically, higher intake of filtered coffee, low-calorie drinks, and other cereals, as well as increased metabolic circulating levels of gamma-tocopherol, were associated with an elevated risk of periodontitis. Conversely, consumption of cheese, white rice, chocolate bars, unsalted peanuts, and higher absolute circulating levels of vitamin C were linked to a reduced risk. Additionally, the study suggests that periodontitis may influence dietary habits, indicating a bidirectional relationship.

**Conclusion:**

This study provides additional evidence of a bidirectional association between dietary factors and periodontitis. It highlights the importance of dietary interventions in the prevention and management of periodontitis. The findings underscore the need for incorporating dietary counseling into periodontal disease management protocols and suggest the potential of personalized dietary strategies for periodontitis patients. Further research is warranted to explore the mechanisms underlying these associations and to confirm these findings in diverse populations.

## 1 Introduction

Periodontitis, a multifaceted inflammatory condition, is precipitated by pathogenic biofilms formed by dental plaque (Könönen et al., 2019). This ailment not only impinges on gingival and skeletal integrity but also compromises alveolar bone health. Manifestations of periodontitis include gingival hemorrhage, development of periodontal pockets, and progressive loss of tissue attachment, and these conditions may ultimately culminate in tooth mobility and subsequent tooth loss (Khajuria et al., 2018). Numerous studies have delineated a variety of risk factors associated with periodontitis, such as lifestyle choices, obesity, metabolic syndrome, and genetic susceptibility (Genco and Borgnakke, 2013). Additionally, nutritional habits play a pivotal role in maintaining holistic health, whereas salubrious dietary patterns are essential for health promotion, and in contrast, deleterious eating habits are linked to an array of chronic pathologies (Noratto et al., 2018).

Various diets were found to be associated with periodontitis in different ways, highlighting the significant impact of nutrition on oral health. One study has found a latent correlation between diet and periodontitis, with their interconnection established through factors beyond diet-dependent inflammatory propensities (Syrjäläinen et al., 2023). For example, vegetarians, compared to non-vegetarians, have lower periodontal pocket depth (PPD) and less gingival bleeding (Staufenbiel et al., 2013). A population cohort study demonstrated a significant association between higher adherence to the DASH (Dietary Approaches to Stop Hypertension)/Mediterranean diets and a reduced incidence of periodontal disease (Altun et al., 2021). Moreover, pro-inflammatory dietary patterns such as red meat, processed meat, etc. were found to be associated with an increased risk of periodontitis (Li et al., 2021; Machado et al., 2021). Among the younger population higher intake of added sugars may increase the risk of periodontitis (Lula et al., 2014). Another study has shown diets containing fewer healthy plant-based (natural foods) foods were associated with an increased risk of periodontitis (Li A. et al., 2023). Research also demonstrated the complicated relationship between diets and periodontitis that a higher intake of whole grains is less likely to lead to periodontitis, but not all plant-based foods are beneficial for periodontal health (Merchant et al., 2006). From a public health perspective, dietary interventions could be a promising non-pharmacological strategy for the prevention of periodontitis (Dommisch et al., 2018). However, further research is required to assess the extent to which macro and micronutrients can influence the initiation and progression of periodontitis (Altun et al., 2021). Additionally, there is limited research on how periodontitis may affect dietary structure and habits.

Aside from dietary habits, research has found antioxidants are also associated with periodontitis. Antioxidants are agents that can effectively inhibit reactive oxygen species (ROS), and by neutralizing the damage caused by oxidative stress, antioxidants provide a therapeutic effect on periodontitis (Sczepanik et al., 2020). Vitamin E, C, and carotenoids represent some of the most accessible dietary antioxidants. Some studies have shown that low serum levels of vitamin C may be considered a risk factor for periodontitis, suggesting dietary insufficiency potentially increases the risk of periodontitis onset (Chapple et al., 2007; Gokhale et al., 2013; Lee et al., 2017; Park et al., 2017; Tada and Miura, 2019; Munday et al., 2020; Assaf and Rabi, 2022), though some reports failed to find this significant association between vitamin C and risk of periodontitis (Ismail et al., 1983; van der Putten et al., 2009). A randomized control trial (RCT) by Singh et al. indicates that supplemental vitamin E can improve periodontal healing and antioxidant defense (Singh et al., 2014). Furthermore, the intake of carotenoids also assists in periodontal treatment (Dodington et al., 2015). Although observational studies have provided evidence linking dietary factors to periodontitis, there are inherent limitations. One major limitation is that periodontitis results from long-term progression, whereas observational studies only capture a snapshot of time and cannot establish a directional association. Also, understanding the associations in epidemiological studies is often challenging due to confounding factors. One robust approach to address these challenges is Mendelian randomization (MR). This method leverages genetic variants as instruments to infer the association between lifelong risk factors (exposures) and diseases (outcomes) (Lawlor et al., 2008). Due to the randomness of meiosis, MR analysis is unaffected by reverse causality or confounding, reducing the bias inherent in traditional observational studies. In this study, we conducted MR analyses to assess the bidirectional associations between diets and periodontitis, aiming to offer new insights for preventing periodontitis.

## 2 Methods

### 2.1 Study design overview

**FIGURE 1:**
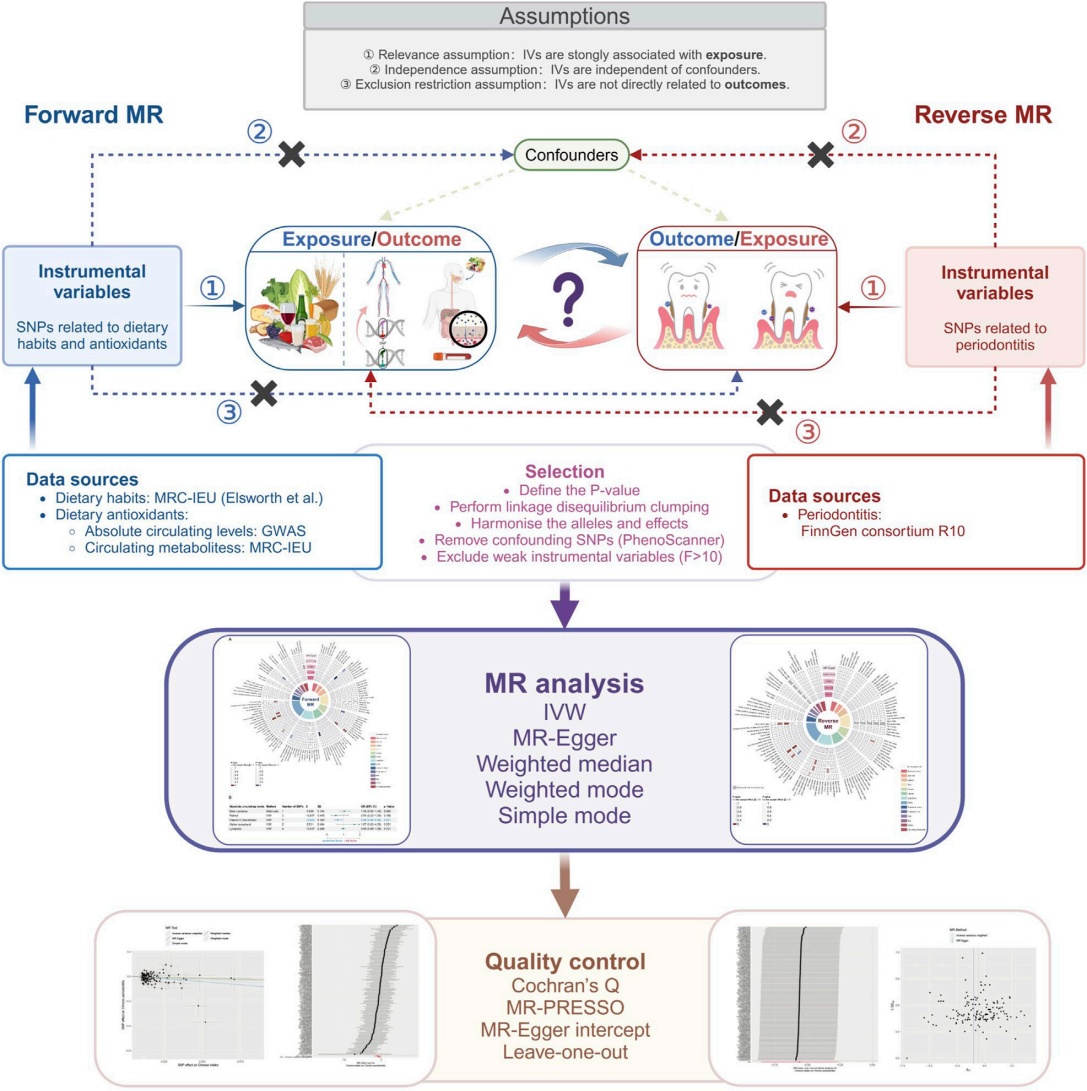
Study design of the association between dietary factors and periodontitis based on Mendelian randomization (MR) assumptions. MR, Mendelian randomization; SNP, single-nucleotide polymorphism; IVs, instrumental variables; F, F statistic indicates the strength of the instrument variables; IVW, inverse-variance weighted; MR-PRESSO, MR pleiotropy residual sum and outlier.

In this study, we explored the bidirectional association between dietary factors and periodontitis using a two-sample Mendelian randomization (MR) approach. The first model examined dietary factors as exposure and periodontitis as the outcome, whereas the second model revered the two having periodontitis as the exposure and dietary habits as the outcome. Both models used filtered single nucleotide polymorphism (SNP) as instrumental variables IV), respectively. The detailed research framework is illustrated in Figure 1. Our study adheres to the STROBE-MR guidelines (Strengthening the Reporting of Observational Studies in Epidemiology using Mendelian Randomization) to ensure comprehensive and transparent reporting (Skrivankova et al., 2021).

### 2.2 Data source

In this study, we obtained summary data for over a hundred dietary habits from the genome-wide association study (GWAS) conducted by the United Kingdom Biobank, an extensive and comprehensive prospective study that recruited over 500,000 participants aged between 40 and 69 years. These participants provided detailed information about their lifestyle and physical measurements, along with genetic and phenotypic data (Sudlow et al., 2015). Detailed information on the collection, cleaning, and analysis of these original data can be found on the United Kingdom Biobank website (http://biobank.ctsu.ox.ac.uk/crystal/label.cgi?id=100052) or the IEU OpenGWAS project (https://gwas.mrcieu.ac.uk/). Dietary-derived antioxidants were divided into two sets: authentic absolute levels measured in blood and their corresponding circulating metabolites quantified as relative concentrations in plasma or serum, as categorized by Luo et al. (Luo et al., 2021). For antioxidants measured at absolute levels, SNPs from five GWAS studies were used to identify alpha-tocopherol (Major et al., 2011), beta-carotene (Hendrickson et al., 2012), lycopene (D’Adamo et al., 2016), retinol (Mondul et al., 2011), and Vitamin C (Zheng et al., 2021). The SNPs data for antioxidant metabolites assessed as metabolic products, including alpha-tocopherol, gamma-tocopherol, carotene, retinol, and Vitamin C, were extracted from the IEU OpenGWAS project database (Elsworth et al., 2020).

Periodontitis data was sourced from the FinnGen project (FinnGen, 2024). It is a large-scale genomics project correlating genetic variants with health data to elucidate disease mechanisms and susceptibility. The project has analyzed over 500,000 samples from the Finnish Biobank to date (Q3 2023), involving multiple institutes such as Finnish research institutions, biobanks, and international industry partners (Kurki et al., 2023). Within the FinnGen consortium, chronic periodontitis diagnosis (encompassing 4784 clinical cases and 272,252 controls of European ancestry) was classified according to the Finnish version of the International Classification of Diseases (ICD).

Comprehensive information on the diets and periodontitis datasets is presented in Supplementary Table S1, with full details available in the cited original public sites.

### 2.3 Selection of instrumental variables

The reliability of association inferences in Mendelian randomization (MR) analyses is primarily contingent upon the appropriate use of instrumental variables (IVs). IVs are generally genetic variations, among which single nucleotide polymorphisms (SNPs) are the most commonly used. SNPs mainly refer to the DNA sequence polymorphism caused by single nucleotide variation at the genomic level. It is ubiquitous in human and animal genomes and accounts for more than 90% of all known polymorphisms in human heritable variation. Therefore, as IVs, SNPs are widely used in Mendelian randomization. To ensure that the three fundamental assumptions of IVs—namely relevance, independence, and exclusion restriction—are met, we meticulously selected SNPs as IVs *via* the subsequent steps.

1) We identified SNPs significantly associated with the exposure (p < 5 × 10^−6^) (in forward model, the SNPs were significantly associated with dietary habits, dietary antioxidants; in reverse model, the SNPs were significantly associated with periodontitis). We conducted linkage disequilibrium analysis to ensure their independence, with parameters set to *R* ^2^ < 0.001 and a clumping distance of 10,000 kb.
2) Subsequently, we harmonized the clumped SNPs data with the assistance of effect allele frequencies, and palindromic SNPs were excluded.
3) We then excluded SNPs potentially related to confounding factors such as smoking status, body mass index, anemia characteristics, fasting glucose (Wang et al., 2022), and educational levels (Baumeister et al., 2022), utilizing the PhenoScanner database (http://www.phenoscanner.medschl.cam.ac.uk/) as a reference (Kamat et al., 2019).
4) To minimize the risk of weak instrument bias, we calculated F-statistics (Chen et al., 2023), as an index of IV strength in MR analysis and SNPs with an F-score less than 10 were excluded.

### 2.4 MR estimates

In this study, we applied five Mendelian randomization (MR) methods to investigate the associations between identified exposures and outcomes, based on β and SE of the IVs meticulously selected as described previously.

1) Inverse Variance Weighted (IVW): A primary MR method that provides an unbiased causal effect estimate by weighting regression outcomes. IVW is effective in detecting causality but is susceptible to pleiotropic effects due to its zero intercept constraint (Burgess et al., 2023).
2) MR-Egger: This method treats MR with multiple IVs as meta-analysis (Bowden et al., 2015), removing the zero intercept constraint and allowing for average pleiotropic effect estimation. It accommodates pleiotropic IVs but is sensitive to weak instrumental variable bias (Burgess and Thompson, 2017).
3) Weighted Median: This approach assigns weights to IVs and can provide consistent estimates even if up to 50% of the IVs are ineffective (Bowden et al., 2016).
4) Weighted Mode: Combines MR estimates by weighting the effects of different genetic variants, using the mode of these results to minimize outlier biases (Hartwig et al., 2017).
5) Simple Mode: An unweighted approach using empirical density for effect estimation (Hemani et al., 2018b).

### 2.5 Sensitivity analysis

Cochran’s Q test was utilized to detect heterogeneity among individual SNP effects in our sensitivity analyses, with a significance level of 0.05 (Greco M et al., 2015). The MR-Egger intercept test was also used to assess the presence of directional pleiotropy by judging whether the intercept term significantly differs from zero (*p* < 0.05) or not (Burgess and Thompson, 2017). Additionally, MR-PRESSO (MR pleiotropy residual sum and outlier) was utilized to detect the presence of horizontal pleiotropy (Verbanck et al., 2018), enabling identifying and excluding outlier SNPs and estimating results after correction. Finally, the “Leave-one-out” method, which involved recalculating and visualizing the overall effect by sequentially omitting each SNP, was applied to examine whether the IVW estimate is driven by a single SNP, allowing us to mitigate bias from single genetic variants (Hemani et al., 2018a).

All statistical analyses were performed using R (version 4.3.2). Using packages such as “MendelianRandomization” “TwoSampleMR”, and “MR-PRESSO”.

## 3 Results

### 3.1 Characteristics of selected SNPs

In this study, we explored a broad spectrum of dietary factors, encompassing 111 distinct dietary habits, alongside five absolute and five metabolic circulating levels of antioxidants, as potential exposures to assess their influence on periodontitis risk. Utilizing data from the PhenoScanner website and the GWAS Catalog, we pinpointed 321 SNPs linked to established periodontitis risk factors, as detailed in Supplementary Table S2. These SNPs were subsequently omitted from our effect analysis. After screening, the number of SNPs for each dietary factor ranged from 1 to 213. Exhaustive details on these instrumental variables (IVs) are provided in Supplementary Table S3. The F-statistics for each identified SNP exceeded the empirical threshold of 10, ranging from 16 to 811.86, suggesting that the evaluation results are less likely to be biased by weak IVs.

Similarly, periodontitis was investigated as an exposure to explore its influence on dietary habits and the circulating metabolic levels of dietary antioxidants (outcome). Due to the significance level of *p* < 5 × 10^−6^ and the harmonization of effect SNPs, the number of SNPs serving as conforming IVs of exposure narrowed down from the original to a range of 7–11 (Supplementary Table S4). In all reverse MR assessments, the F-statistics for each ranged from 20.87 to 24.72, indicating a reduced likelihood of evaluation results being skewed by weak IVs. Because SNPs for absolute circulating levels of antioxidants from published reports were only used as exposures, we did not assess the impact of periodontitis on these (Luo et al., 2021; Miao et al., 2022; Li H. et al., 2023).

### 3.2 The impact of dietary factors on periodontitis

**FIGURE 2:**
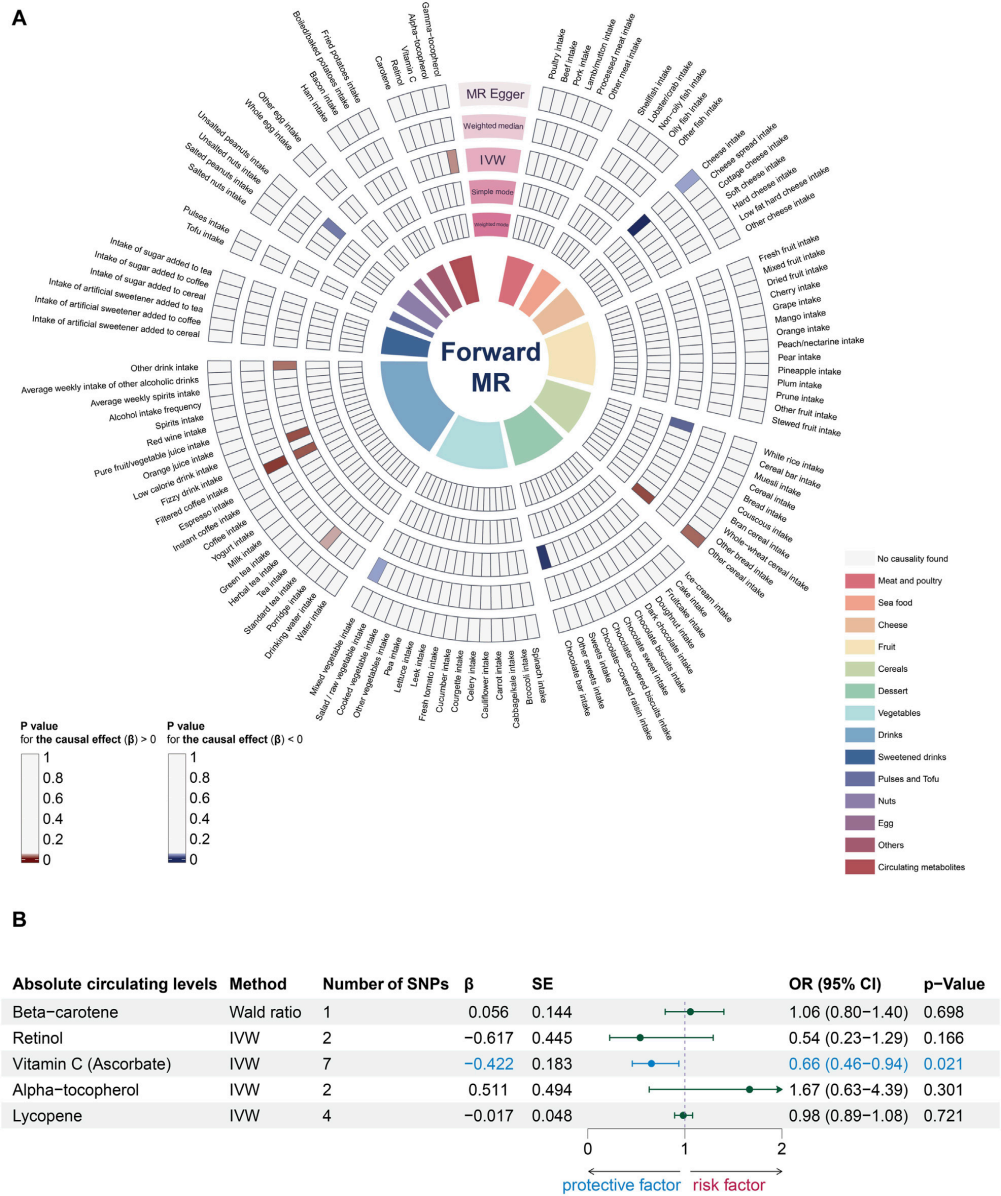
The impact of dietary factors on periodontitis. **(A)** The ring heatmap shows a summary of the effects of dietary factors on the risk of periodontitis, displaying all results from five different methods. When *p*-value > 0.05, red color block indicates risk factors (positive correlation) and blue color block indicates protective factors (negative correlation). **(B)** The forest plot shows the effects of dietary antioxidants at absolute circulating levels on the risk of periodontitis. MR, Mendelian randomization; IVW, inverse-variance weighted; SNP, single-nucleotide polymorphism; β, effect size; SE, standard error; OR, odds ratio; CI, confidence interval.

We utilized five different MR methods to analyze the impact of various dietary factors on the risk of periodontitis, with the summarized results depicted in Figure 2 and detailed data in Supplementary Table S5. Specifically, based on the IVW method, we identified ten potential association between dietary factors and the risk of periodontitis. Certain dietary factors, such as the intake of other cereals (Odds Ratio [OR] = 7.95, 95% Confidence Interval [CI] = 1.49-42.52, *p*-value = 0.02), filtered coffee (OR = 1.42, 95% CI = 1.06-1.89, *p*-value = 0.02), low-calorie drinks (OR = 1.57, 95% CI = 1.09-2.27, *p*-value = 0.02), other drinks (OR = 3.25, 95% CI = 1.15-9.17, *p*-value = 0.03), and the metabolic circulating levels of gamma-tocopherol (OR = 2.10, 95% CI = 1.06-4.16, *p*-value = 0.03), were found to be associated with an increased risk of periodontitis. Conversely, the intake of cheese (OR = 0.57, 95% CI = 0.43-0.77, *p*-value = 0.0002), white rice (OR = 0.42, 95% CI = 0.20-0.88, *p*-value = 0.02), chocolate bars (OR = 0.46, 95% CI = 0.27-0.80, *p*-value = 0.005), unsalted peanuts (OR = 0.29, 95% CI = 0.09-0.88, *p*-value = 0.03), and the absolute circulating level of vitamin C (OR = 0.66, 95% CI = 0.46-0.94, *p*-value = 0.02) were associated with a decreased risk of the disease.

### 3.3 The impact of periodontitis on dietary habits and dietary antioxidants

**FIGURE 3:**
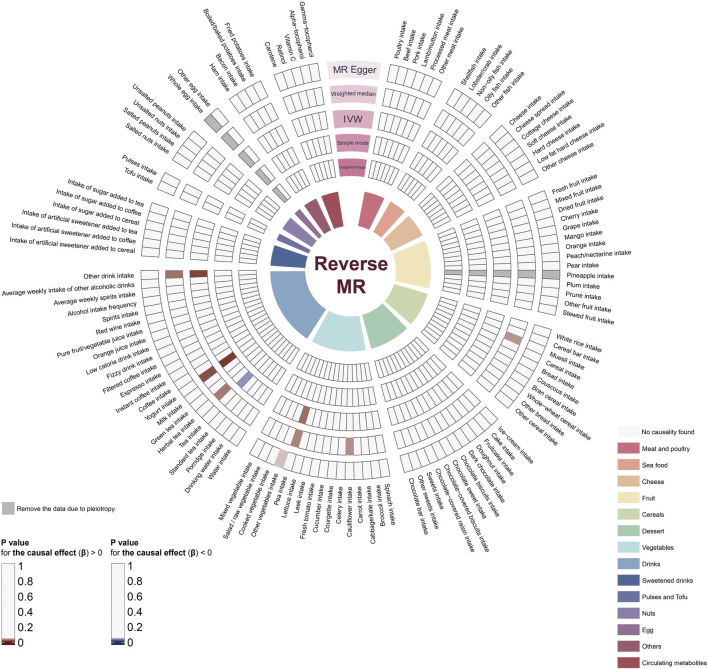
The impact of periodontitis on dietary habits and dietary antioxidants. The ring heatmap shows a summary of the effects of periodontitis on dietary habits, displaying all results from five different methods. When *p*-value > 0.05, red color block indicates risk factors (positive correlation) and blue color block indicates protective factors (negative correlation). MR, Mendelian randomization; IVW, inverse-variance weighted.

Similarly, we employed five distinct MR methods to assess the influence of periodontitis on dietary habits and the metabolic levels of dietary antioxidants. The aggregated results are illustrated in Figure 3, with exhaustive details provided in Supplementary Table S6. Specifically, based on the IVW method, we identified four dietary habits potentially affected by periodontitis. Our findings suggest that periodontitis is likely associated with an increased intake of peas (OR = 0.99, 95% CI = 0.97-1.00, *p*-value = 0.04), yogurt (OR = 1.05, 95% CI = 1.01-1.09, *p*-value = 0.01), and other drinks (OR = 1.02, 95% CI = 1.00-1.03, *p*-value = 0.01), and conversely, a decreased consumption of tea (OR = 0.99, 95% CI = 0.97-1.00, *p*-value = 0.04).

### 3.4 Sensitivity analysis

In the forward MR analysis about the impact of dietary factors on periodontitis, IVW, and MR-Egger tests did not reveal significant heterogeneity (Cochran’s Q *p*-values >0.05) (Supplementary Table S7). Additionally, no direct or horizontal pleiotropy was detected by the MR-Egger intercept test (MR Egger *p*-values >0.05) and the MR-PRESSO global test (after removing outliers), respectively (Supplementary Table S7 and Supplementary Table S9). Moreover, we constructed scatter plots, forest plots, leave-one-out analysis, and funnel plots to visualize the results of sensitivity analyses (Supplementary Figures S1–S10), reinforced the findings. The scatter plots, depicting effect estimates of each SNP calculated using 5 MR methods and their line fitting, provide an intuitive understanding of the effects of exposures on outcomes. Forest plots, visualizing the main results of MR analysis, clearly exhibit the effects of exposures on outcomes. The leave-one-out analysis indicated no single SNP strongly influenced the overall effect. Lastly, funnel plots’ symmetry and lack of skewness confirm the absence of significant bias in the selected SNPs. Similarly, this series of sensitivity analysis procedures are used for the reverse MR analysis model about the impact of periodontitis on dietary habits and dietary antioxidants. Cochran’s Q test did not reveal significant heterogeneity among these IVs (Supplementary Table S8). The MR-Egger intercept test indicated the presence of pleiotropy in two dietary habits’ IVs, leading to their exclusion, while MR-PRESSO global test did not detect any pleiotropy (after removing outliers) (Supplementary Tables S8, S10). Also, scatter plots, forest plots, leave-one-out analysis, and funnel plots were constructed to visually represent the results of sensitivity analyses to strengthen these findings (Supplementary Figures S11–S14).

Combining the results of all MR methods, we observed that there was no association meeting the criteria of *p* < 0.05 simultaneously across all 5 MR methods (Figure 2; Figure 3). Therefore, we utilized data analyzed using the other four methods to complement the assessment of the effect using IVW (the gold standard). Since the direction of β estimates from all the other MR methods in the aforementioned results remained largely consistent with the IVW results (with no more than two conflicting outcomes), we ruled out potential interference arising from different methods.

## 4 Discussion

This study employed a comprehensive Mendelian Randomization (MR) analysis to explore the bidirectional association between diets and periodontitis. This study has found multiple dietary habits to be associated with periodontitis and *vice versa*.

### 4.1 Impact of dietary factors on periodontitis

The forward MR analyse model, focusing on dietary factors as exposures, provides intriguing insights into the associations between specific dietary habits and periodontitis risk. Our analysis revealed several dietary factors that potentially influence periodontitis risk. Specifically, the consumption of filtered coffee, low-calorie drinks, other drinks, other cereals, and the metabolic circulating levels of gamma-tocopherol were associated with an increased risk of periodontitis. These raise questions about the potential mechanisms underlying these associations. Factors such as the bioavailability of certain nutrients, additives in low-calorie drinks, and the inflammatory impact of specific cereals may contribute to these outcomes. The findings align with previous research linking pro-inflammatory dietary patterns to an increased risk of periodontitis, suggesting the pro-inflammatory potential of certain dietary choices (Li et al., 2021). Additionally, direct evidence on the use of Vitamin E (gamma-tocopherol) supplements for the treatment of periodontitis remains limited. This study provides evidence at the metabolic level that Vitamin E might be a risk factor for periodontitis through MR analysis, offering a reference. Notably, the confidence intervals for ‘other cereals’ are exceptionally wide. Upon reviewing the original 24-h dietary recall questionnaire from the primary website (https://biobank.ndph.ox.ac.uk/ukb/refer.cgi?id=118240&nl=1), we speculate that this may be due to the broad concept of ‘other’ in the questionnaire, leading to wider confidence intervals than typically observed. Conversely, the intake of cheese, white rice, chocolate bars, unsalted peanuts, and the absolute circulating level of vitamin C were associated with a decreased risk of the disease. The lack of significant heterogeneity or pleiotropy in our sensitivity analyses strengthens the validity of these associations. This is in line with the notion that certain foods and antioxidants possess anti-inflammatory properties, potentially mitigating periodontal inflammation (Dodington et al., 2015; Altun et al., 2021). It is worth noting that existing observational studies primarily focus on overall dietary patterns. Our study emphasizes analyzing the specific dietary habits and their effect on the risk of periodontitis. More well-designed and controlled studies are needed to determine the effectiveness.

### 4.2 Impact of periodontitis on dietary habits and antioxidants metabolic levels

Interestingly, the reverse MR analyse model of our study, where periodontitis is considered the exposure, suggests that periodontitis may influence dietary choices, a relatively unexplored dimension in periodontal research. The increased intake of peas, yogurt, and other drinks among individuals with periodontitis might reflect compensatory dietary adaptations due to the disease’s impact on oral health and masticatory function. Conversely, the reduced tea intake observed in periodontitis patients could be attributed to changes in taste preferences or avoidance of certain foods that might exacerbate oral discomfort. However, the potential influence of these dietary changes on periodontal health remains to be further investigated.

### 4.3 Sensitivity analysis

The sensitivity analyses conducted in this study strengthen the findings’ robustness. The absence of significant heterogeneity and pleiotropy in most analyses suggests that confounding factors or outliers do not unduly influence the results. The use of multiple MR methods and thorough sensitivity assessments enhances the reliability of the relationship inferences drawn from the study.

### 4.4 Potential implications and interventions

Understanding the bidirectional associations between dietary factors and periodontitis has important implications for interventions. The identified dietary patterns associated with increased or decreased risk offer potential targets for dietary interventions. Incorporating dietary counseling into periodontal disease management protocols is warranted, emphasizing the potential benefits of personalized dietary strategies. Further research is essential to explore the mechanistic links between specific dietary components and periodontitis and to validate these findings across diverse populations.

### 4.5 Limitations and future research

While our study provides valuable insights, there are some limitations. Despite the robustness of MR, relying on observational data from GWAS cannot establish a definitive causality due to linkage disequilibrium or population stratification. The potential for residual confounding and the influence of unmeasured factors cannot be completely excluded. Moreover, the generalizability of our findings might be limited by the ethnic and demographic characteristics of the populations in the GWAS datasets used. Future research should aim to replicate these findings in diverse populations and to validate these findings through prospective studies or randomized controlled trials. Furthermore, investigating the underlying mechanisms through which diet influences periodontal health, and *vice versa*, would contribute significantly to the understanding of periodontitis pathophysiology.

## 5 Conclusion

In conclusion, our study underscores the bidirectional relationship between diets and periodontitis, highlighting the potential of dietary modifications as a non-pharmacological intervention for the prevention and management of periodontitis. These findings emphasize the importance of incorporating dietary counseling into periodontal disease management protocols and warrant further research into personalized dietary strategies for periodontitis patients.

## Data Availability

The original contributions presented in the study are included in the article/Supplementary Material, further inquiries can be directed to the corresponding authors.
