# Supplementary material for: The association between diets and periodontitis: a bidirectional two-sample Mendelian randomization study
Source: Front Genet. 2024 May 31;15:1398101. doi: 10.3389/fgene.2024.1398101 (PMC11176517; doi:10.3389/fgene.2024.1398101)
Supplement: Supplementary file 2 [file Table2.DOCX]

**Additional File S2**

**The association between diets and periodontitis: a bidirectional two-sample Mendelian randomization study**

# Xiaoyu Yang^1,2,3†^, Jingchan Wang^1,2,3†^, Houlin Hong^4^, Xing Feng^1,2,3^, Xiumei Zhang^5*^, Jinlin Song^1,2,3*^

**Supplemental Fig.** **1** Sensitivity analysis of MR estimates on the association between dietary factors (other cereal intake) and periodontitis.

**Supplemental Fig.** **2** Sensitivity analysis of MR estimates on the association between dietary factors (filtered coffee intake) and periodontitis.

**Supplemental Fig.** **3** Sensitivity analysis of MR estimates on the association between dietary factors (low calorie drink intake) and periodontitis.

**Supplemental Fig. 4** Sensitivity analysis of MR estimates on the association between dietary factors (other drink intake) and periodontitis.

**Supplemental Fig.** **5** Sensitivity analysis of MR estimates on the association between dietary factors (metabolic circulation levels of gamma-tocopherol) and periodontitis.

**Supplemental Fig. 6** Sensitivity analysis of MR estimates on the association between dietary factors (cheese intake) and periodontitis.

**Supplemental Fig.** **7** Sensitivity analysis of MR estimates on the association between dietary factors (white rice intake) and periodontitis.

**Supplemental Fig.** **8** Sensitivity analysis of MR estimates on the association between dietary factors (chocolate bar intake) and periodontitis.

**Supplemental Fig. 9** Sensitivity analysis of MR estimates on the association between dietary factors (unsalted peanuts intake) and periodontitis.

**Supplemental Fig. 10** Sensitivity analysis of MR estimates on the association between dietary factors (absolute circulating level of vitamin C) and periodontitis.

**Supplemental Fig.** **11** Sensitivity analysis of MR estimates on the association between periodontitis and pea intake.

**Supplemental Fig. 12** Sensitivity analysis of MR estimates on the association between periodontitis and yogurt intake.

**Supplemental Fig. 13** Sensitivity analysis of MR estimates on the association between periodontitis and other drink intake.

**Supplemental Fig. 14** Sensitivity analysis of MR estimates on the association between periodontitis and tea intake.


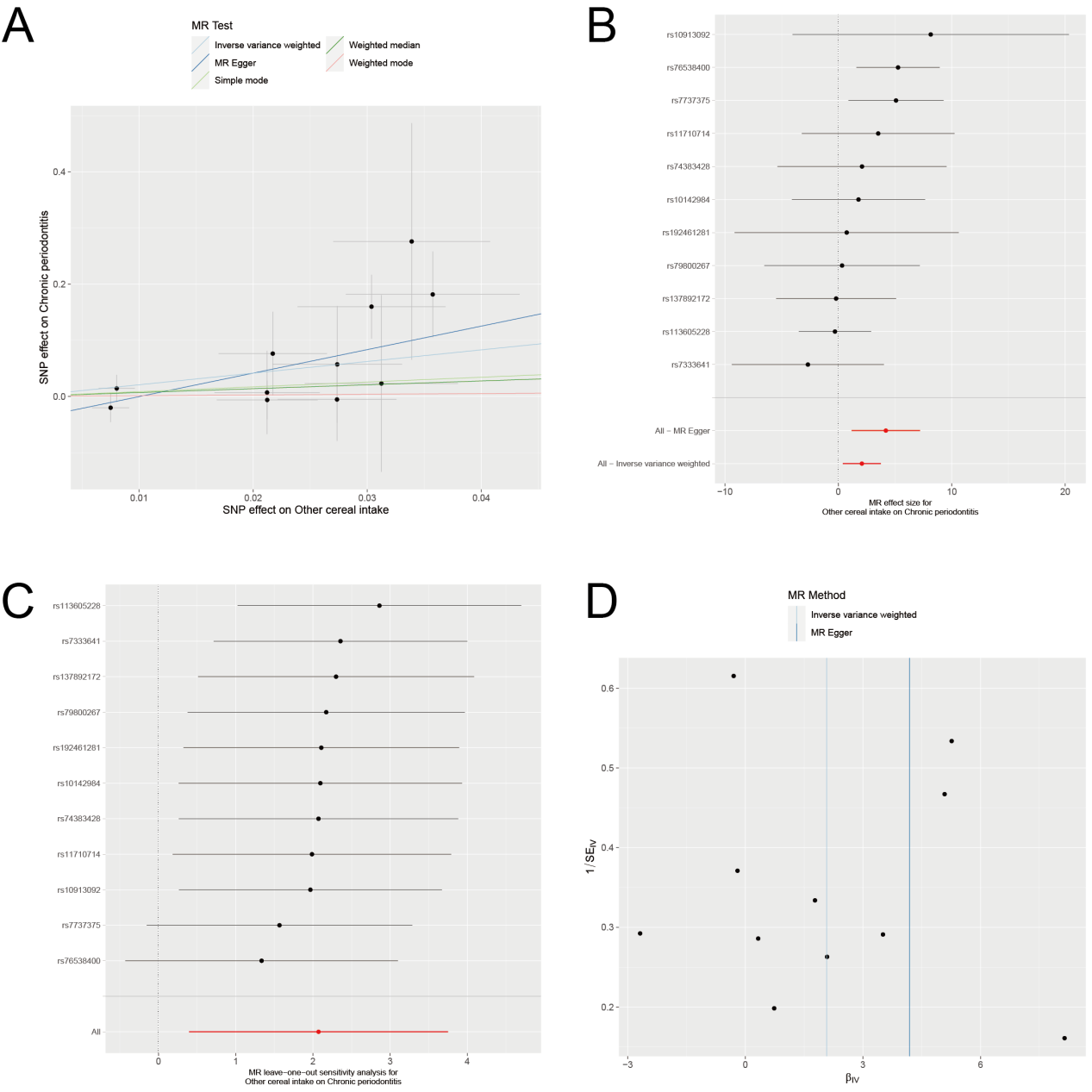


**Supplemental Fig.** **1** **Sensitivity analysis of MR estimates on the association between dietary factors (other cereal intake) and periodontitis. (A)** Scatter Plot: Displays the associations between SNPs and outcomes versus SNPs and exposures, providing effect estimates for individual variants. Different colored lines represent regression slopes fitted by various MR methods. **(B)** Forest Plot: Shows the effect estimate of each SNP, accompanied by 95% confidence intervals. **(C)** Leave-One-Out Analysis: Recalculates effect estimates after sequentially excluding each SNP to determine if a particular SNP significantly influences the association. **(D)** Funnel Plot: Plots estimates against their precision, assessing potential data asymmetry. MR, mendelian randomization; SNP, single-nucleotide polymorphism; β, effect size; SE, standard error.


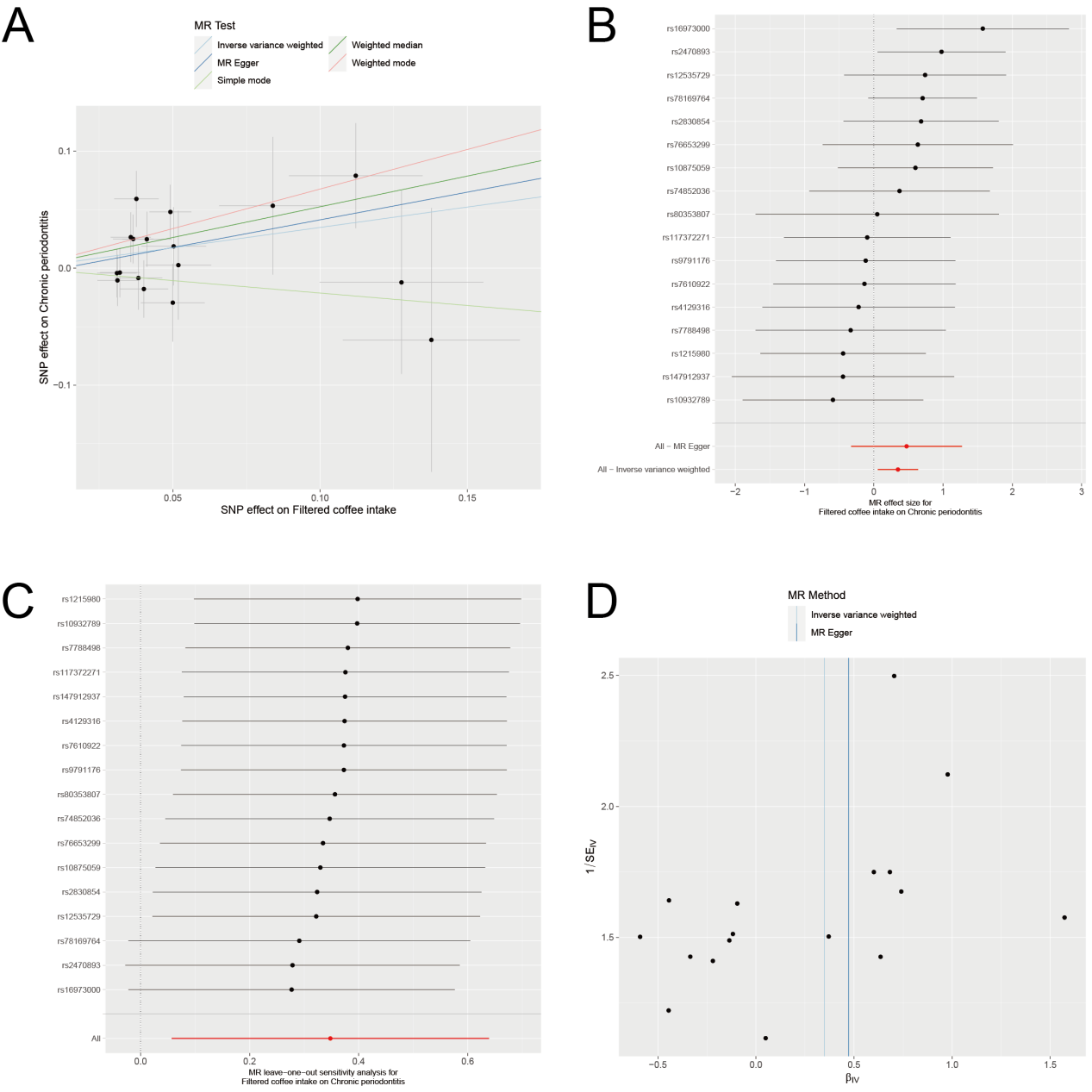


**Supplemental Fig.** **2** **Sensitivity analysis of MR estimates on the association between dietary factors (filtered coffee intake) and periodontitis.** **(A)** Scatter Plot: Displays the associations between SNPs and outcomes versus SNPs and exposures, providing effect estimates for individual variants. Different colored lines represent regression slopes fitted by various MR methods. **(B)** Forest Plot: Shows the effect estimate of each SNP, accompanied by 95% confidence intervals. **(C)** Leave-One-Out Analysis: Recalculates effect estimates after sequentially excluding each SNP to determine if a particular SNP significantly influences the association. **(D)** Funnel Plot: Plots estimates against their precision, assessing potential data asymmetry. MR, mendelian randomization; SNP, single-nucleotide polymorphism; β, effect size; SE, standard error.


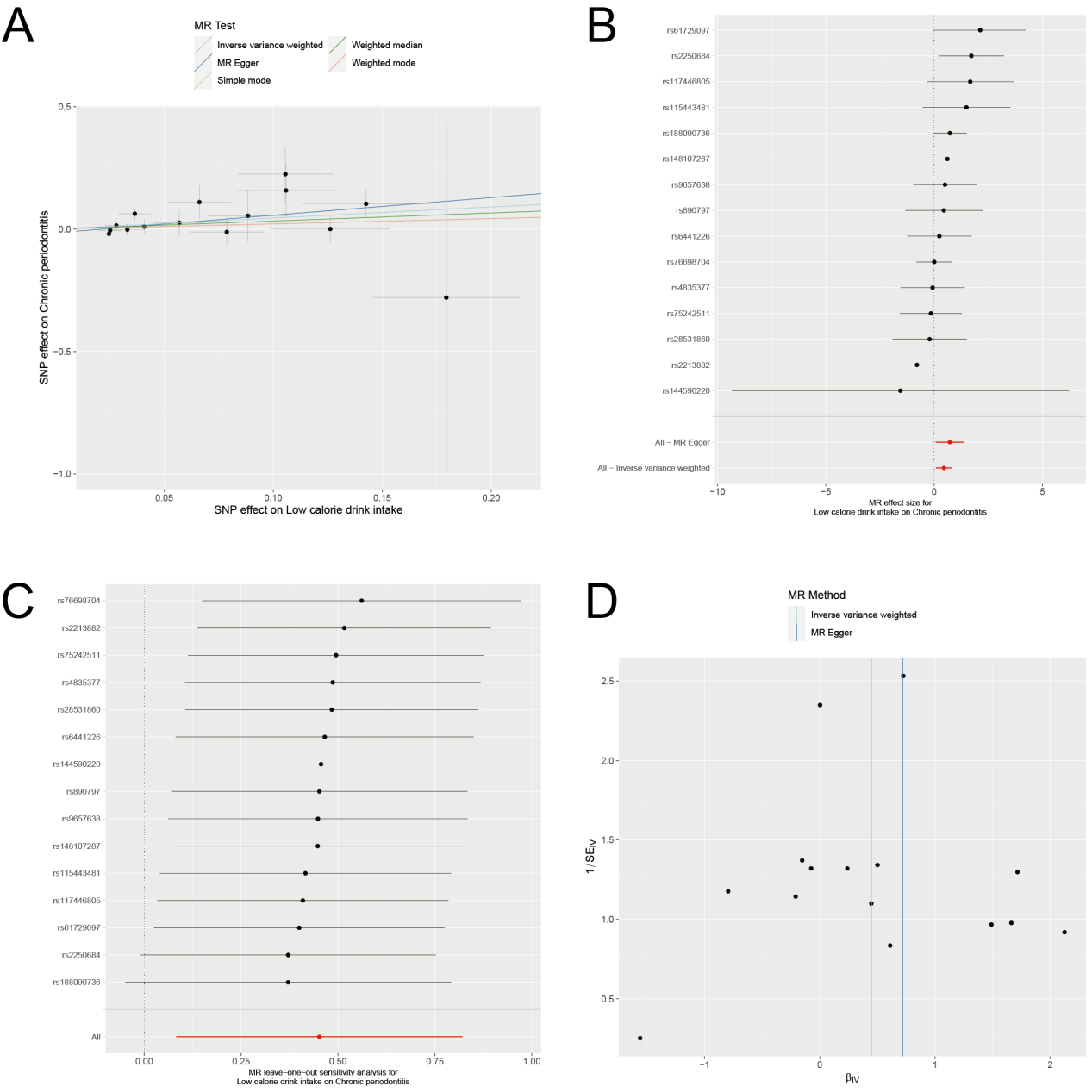


**Supplemental Fig.** **3** **Sensitivity analysis of MR estimates on the association between dietary factors (low calorie drink intake) and periodontitis. (A)** Scatter Plot: Displays the associations between SNPs and outcomes versus SNPs and exposures, providing effect estimates for individual variants. Different colored lines represent regression slopes fitted by various MR methods. **(B)** Forest Plot: Shows the effect estimate of each SNP, accompanied by 95% confidence intervals. **(C)** Leave-One-Out Analysis: Recalculates effect estimates after sequentially excluding each SNP to determine if a particular SNP significantly influences the association. **(D)** Funnel Plot: Plots estimates against their precision, assessing potential data asymmetry. MR, mendelian randomization; SNP, single-nucleotide polymorphism; β, effect size; SE, standard error.


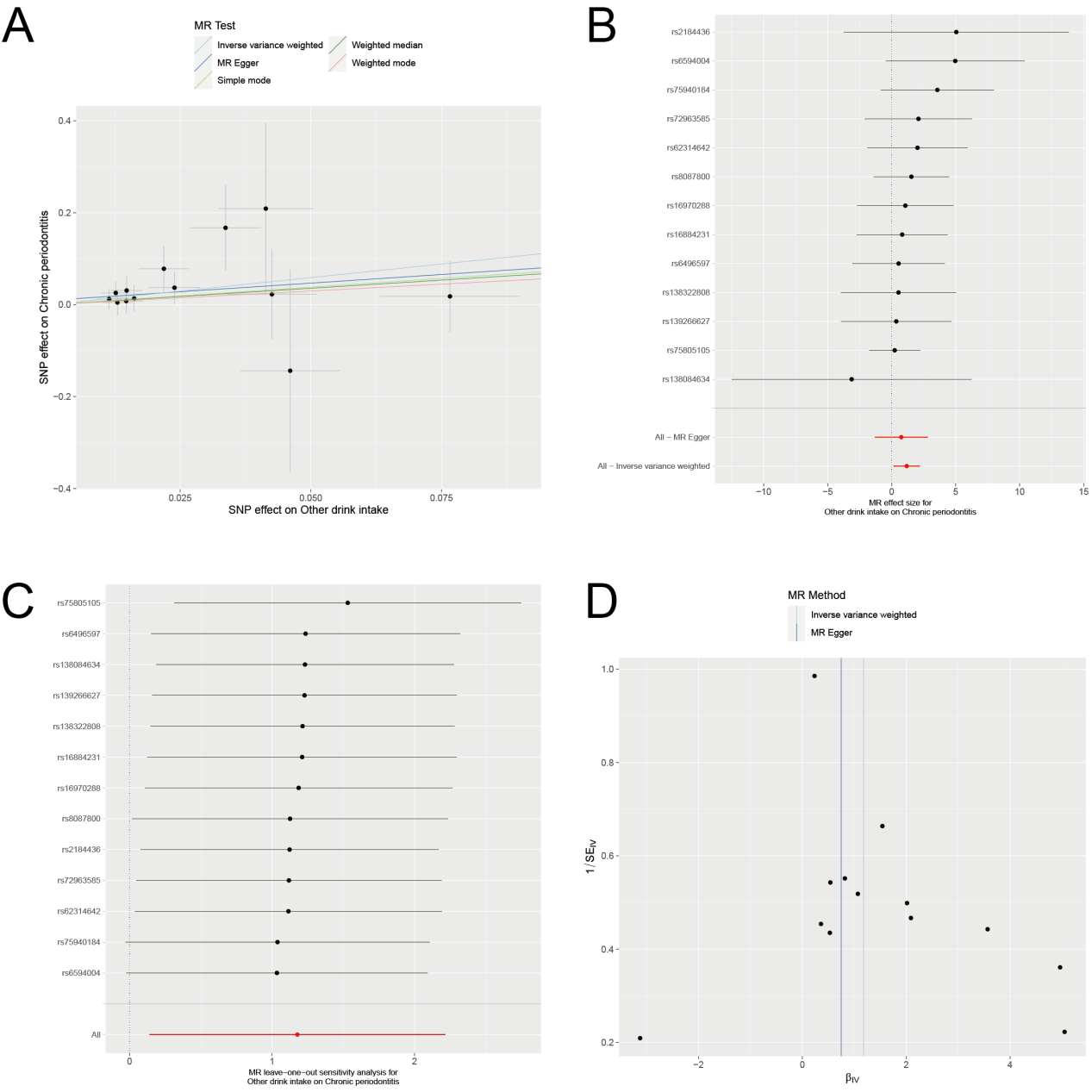


**Supplemental Fig. 4** **Sensitivity analysis of MR estimates on the association between dietary factors (other drink intake) and periodontitis. (A)** Scatter Plot: Displays the associations between SNPs and outcomes versus SNPs and exposures, providing effect estimates for individual variants. Different colored lines represent regression slopes fitted by various MR methods. **(B)** Forest Plot: Shows the effect estimate of each SNP, accompanied by 95% confidence intervals. **(C)** Leave-One-Out Analysis: Recalculates effect estimates after sequentially excluding each SNP to determine if a particular SNP significantly influences the association. **(D)** Funnel Plot: Plots estimates against their precision, assessing potential data asymmetry. MR, mendelian randomization; SNP, single-nucleotide polymorphism; β, effect size; SE, standard error.


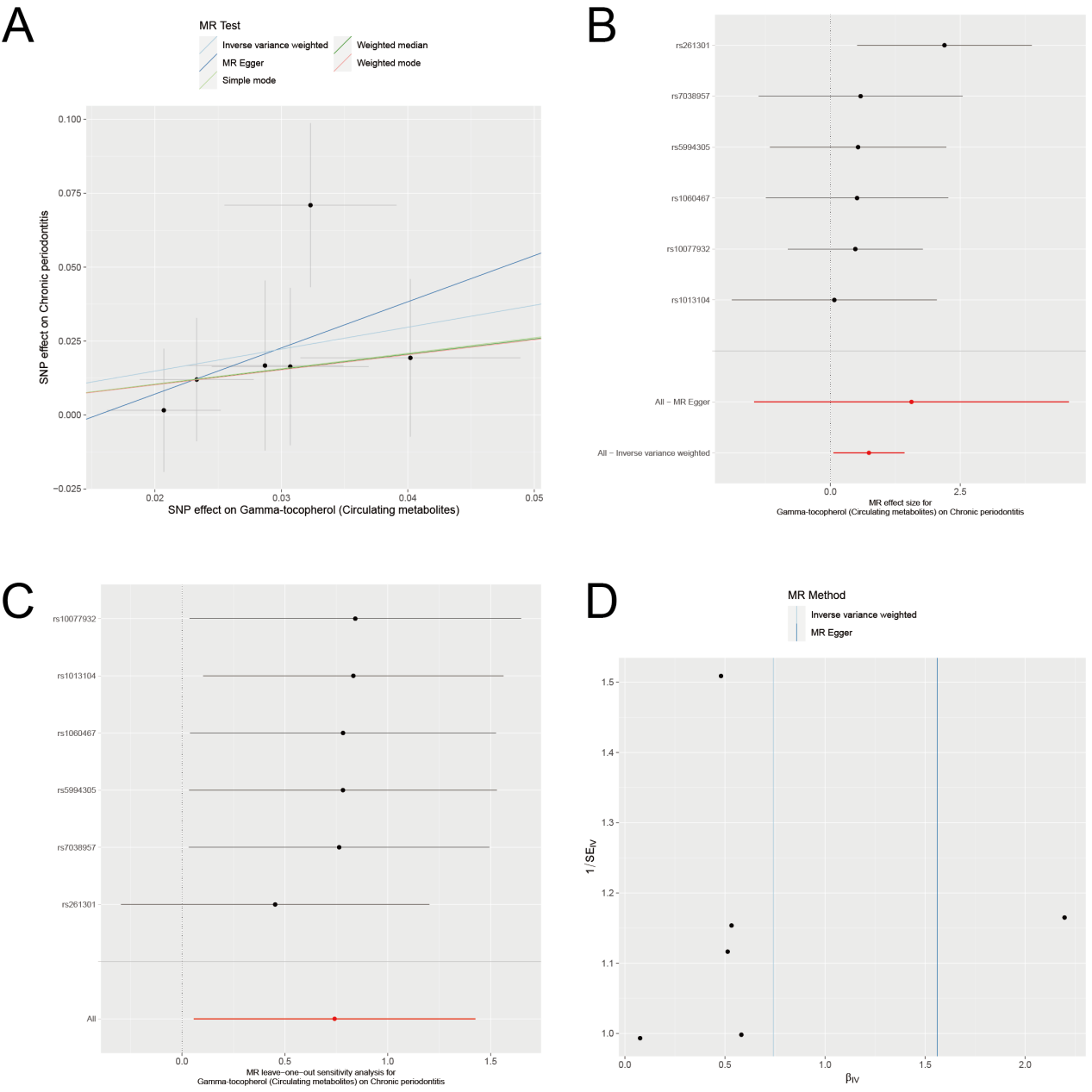


**Supplemental Fig.** **5** **Sensitivity analysis of MR estimates on the association between dietary factors (metabolic circulation levels of gamma-tocopherol) and periodontitis. (A)** Scatter Plot: Displays the associations between SNPs and outcomes versus SNPs and exposures, providing effect estimates for individual variants. Different colored lines represent regression slopes fitted by various MR methods. **(B)** Forest Plot: Shows the effect estimate of each SNP, accompanied by 95% confidence intervals. **(C)** Leave-One-Out Analysis: Recalculates effect estimates after sequentially excluding each SNP to determine if a particular SNP significantly influences the association. **(D)** Funnel Plot: Plots estimates against their precision, assessing potential data asymmetry. MR, mendelian randomization; SNP, single-nucleotide polymorphism; β, effect size; SE, standard error.


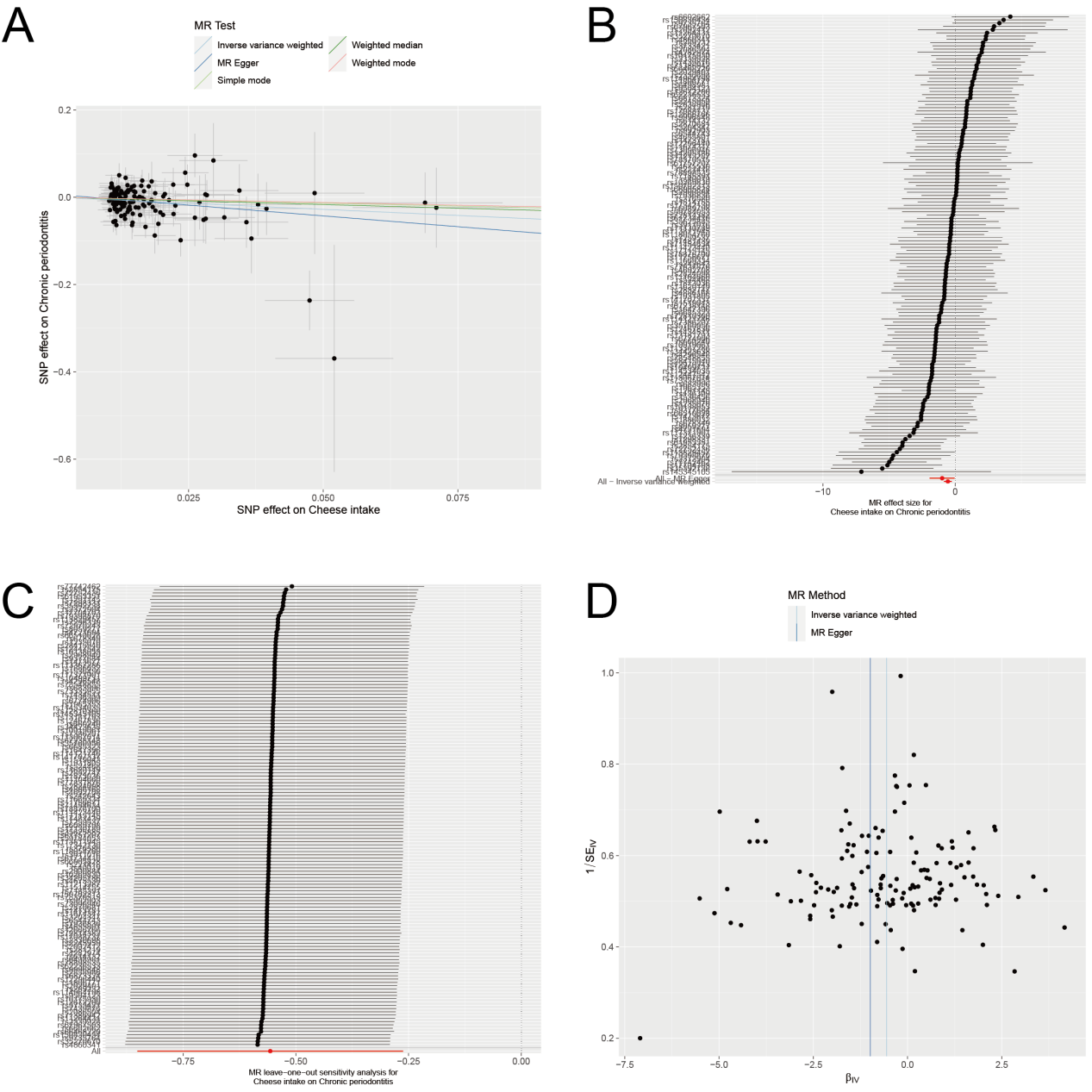


**Supplemental Fig. 6** **Sensitivity analysis of MR estimates on the association between dietary factors (cheese intake) and periodontitis. (A)** Scatter Plot: Displays the associations between SNPs and outcomes versus SNPs and exposures, providing effect estimates for individual variants. Different colored lines represent regression slopes fitted by various MR methods. **(B)** Forest Plot: Shows the effect estimate of each SNP, accompanied by 95% confidence intervals. **(C)** Leave-One-Out Analysis: Recalculates effect estimates after sequentially excluding each SNP to determine if a particular SNP significantly influences the association. **(D)** Funnel Plot: Plots estimates against their precision, assessing potential data asymmetry. MR, mendelian randomization; SNP, single-nucleotide polymorphism; β, effect size; SE, standard error.


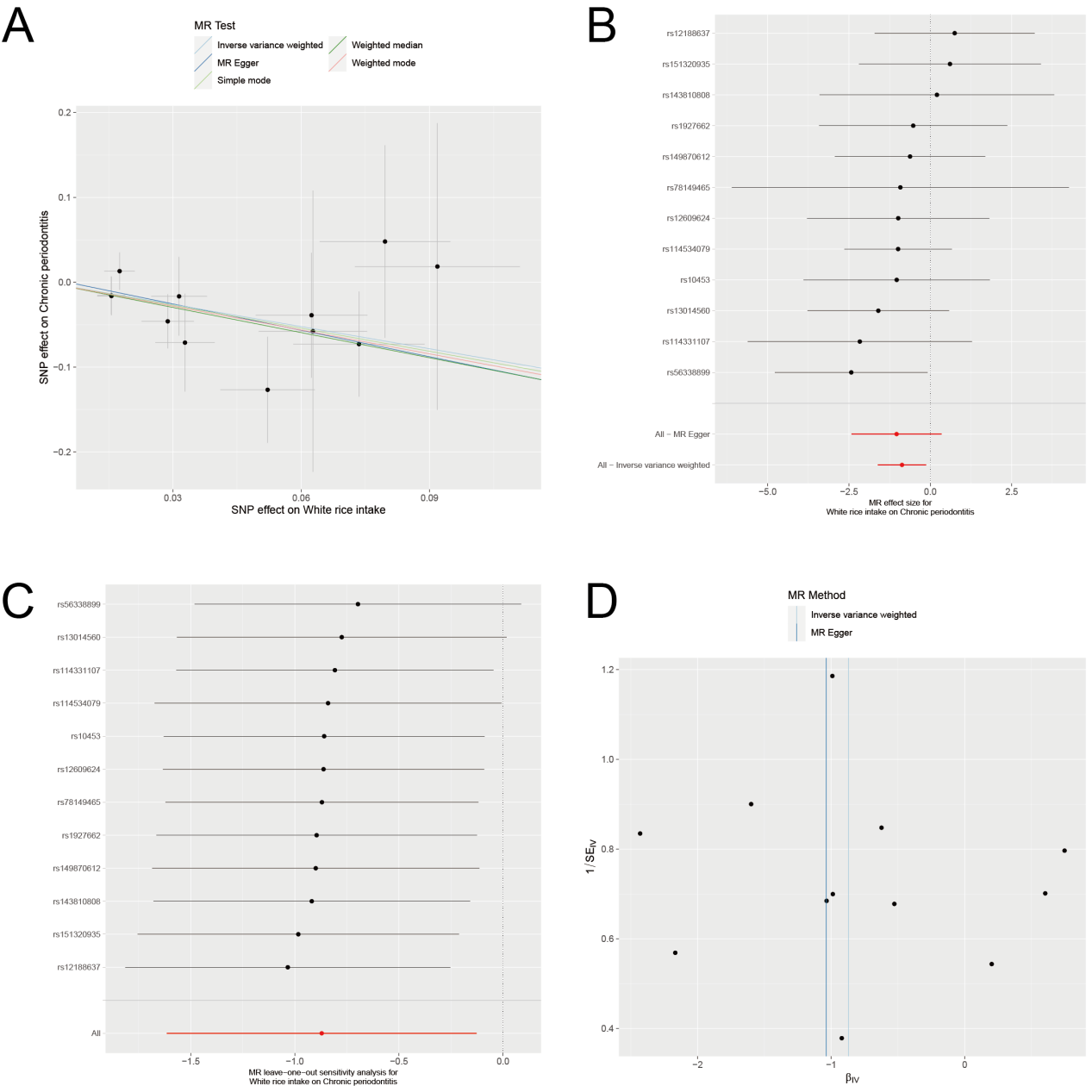


**Supplemental Fig.** **7** **Sensitivity analysis of MR estimates on the association between dietary factors (white rice intake) and periodontitis. (A)** Scatter Plot: Displays the associations between SNPs and outcomes versus SNPs and exposures, providing effect estimates for individual variants. Different colored lines represent regression slopes fitted by various MR methods. **(B)** Forest Plot: Shows the effect estimate of each SNP, accompanied by 95% confidence intervals. **(C)** Leave-One-Out Analysis: Recalculates effect estimates after sequentially excluding each SNP to determine if a particular SNP significantly influences the association. **(D)** Funnel Plot: Plots estimates against their precision, assessing potential data asymmetry. MR, mendelian randomization; SNP, single-nucleotide polymorphism; β, effect size; SE, standard error.


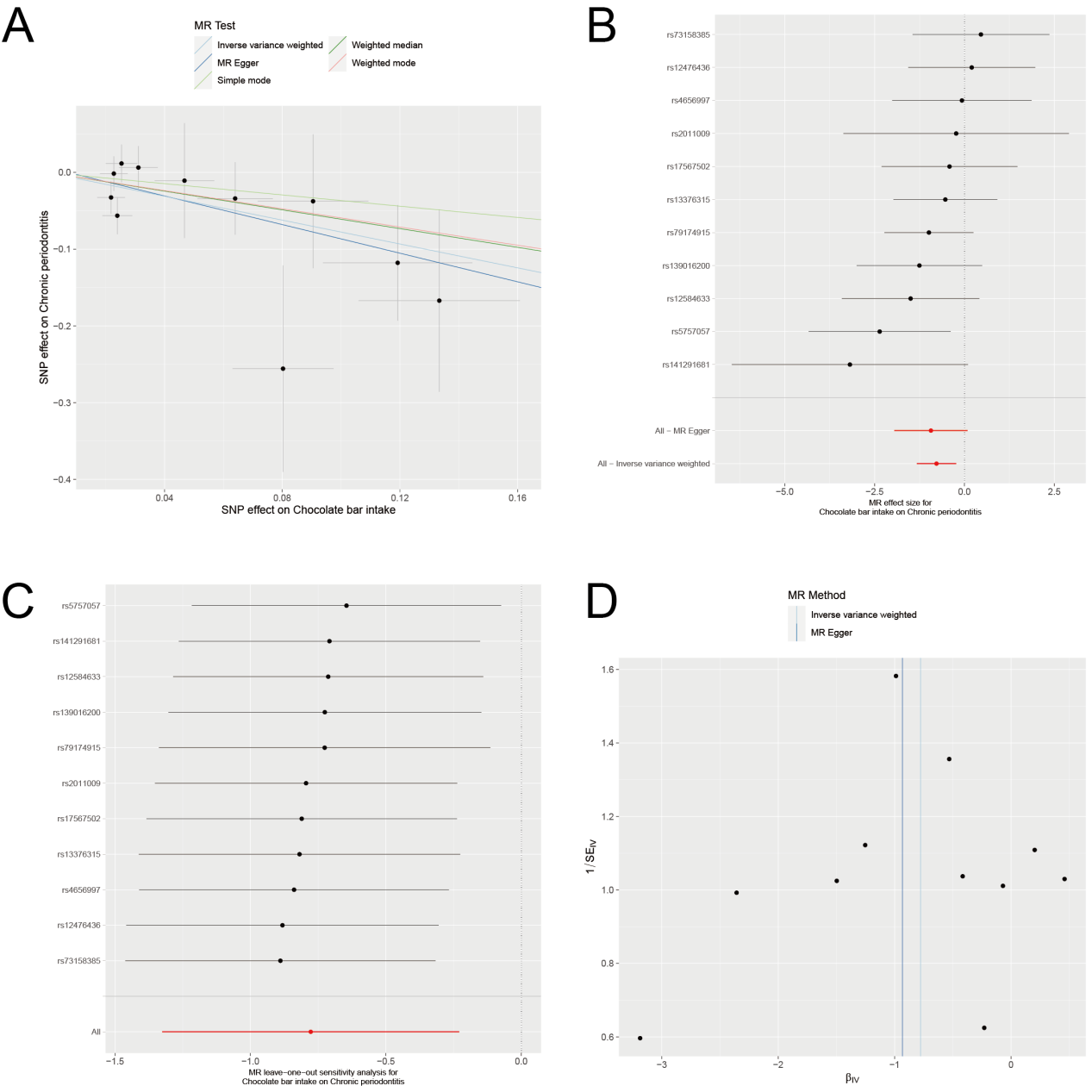


**Supplemental Fig.** **8** **Sensitivity analysis of MR estimates on the association between dietary factors (chocolate bar intake) and periodontitis. (A)** Scatter Plot: Displays the associations between SNPs and outcomes versus SNPs and exposures, providing effect estimates for individual variants. Different colored lines represent regression slopes fitted by various MR methods. **(B)** Forest Plot: Shows the effect estimate of each SNP, accompanied by 95% confidence intervals. **(C)** Leave-One-Out Analysis: Recalculates effect estimates after sequentially excluding each SNP to determine if a particular SNP significantly influences the association. **(D)** Funnel Plot: Plots estimates against their precision, assessing potential data asymmetry. MR, mendelian randomization; SNP, single-nucleotide polymorphism; β, effect size; SE, standard error.


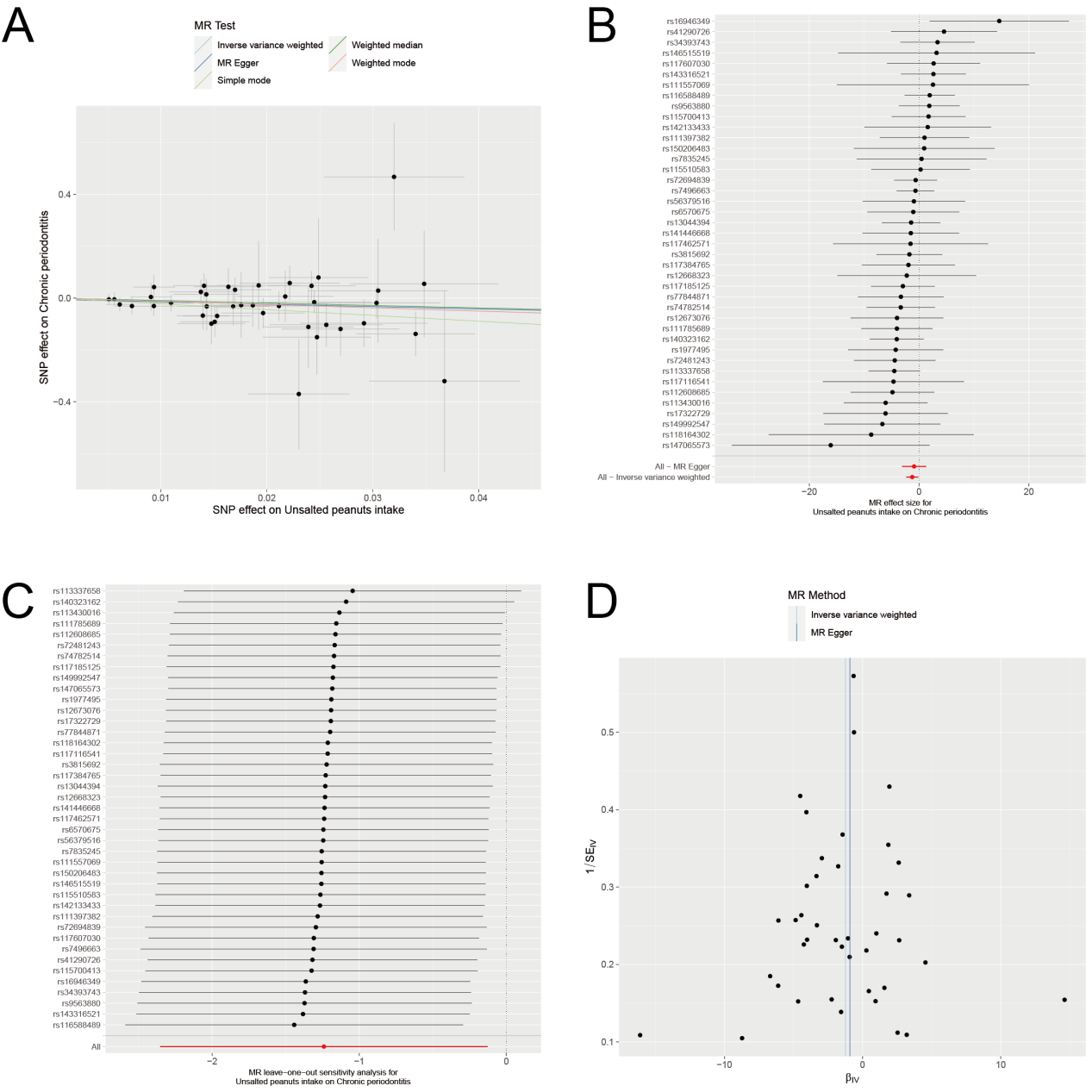


**Supplemental Fig. 9** **Sensitivity analysis of MR estimates on the association between dietary factors (unsalted peanuts intake) and periodontitis. (A)** Scatter Plot: Displays the associations between SNPs and outcomes versus SNPs and exposures, providing effect estimates for individual variants. Different colored lines represent regression slopes fitted by various MR methods. **(B)** Forest Plot: Shows the effect estimate of each SNP, accompanied by 95% confidence intervals. **(C)** Leave-One-Out Analysis: Recalculates effect estimates after sequentially excluding each SNP to determine if a particular SNP significantly influences the association. **(D)** Funnel Plot: Plots estimates against their precision, assessing potential data asymmetry. MR, mendelian randomization; SNP, single-nucleotide polymorphism; β, effect size; SE, standard error.


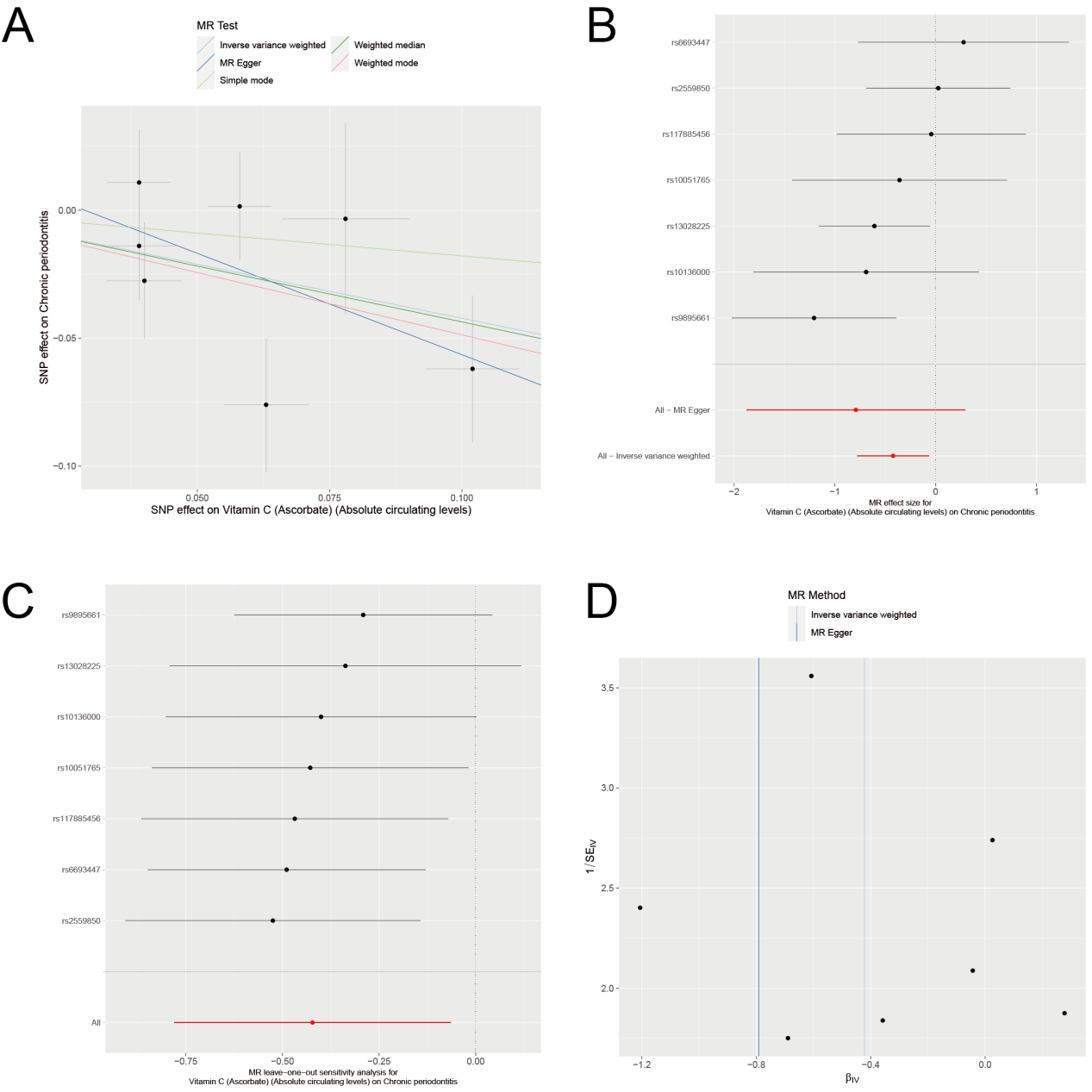


**Supplemental Fig. 10** **Sensitivity analysis of MR estimates on the association between dietary factors (absolute circulating level of vitamin C) and periodontitis. (A)** Scatter Plot: Displays the associations between SNPs and outcomes versus SNPs and exposures, providing effect estimates for individual variants. Different colored lines represent regression slopes fitted by various MR methods. **(B)** Forest Plot: Shows the effect estimate of each SNP, accompanied by 95% confidence intervals. **(C)** Leave-One-Out Analysis: Recalculates effect estimates after sequentially excluding each SNP to determine if a particular SNP significantly influences the association. **(D)** Funnel Plot: Plots estimates against their precision, assessing potential data asymmetry. MR, mendelian randomization; SNP, single-nucleotide polymorphism; β, effect size; SE, standard error.


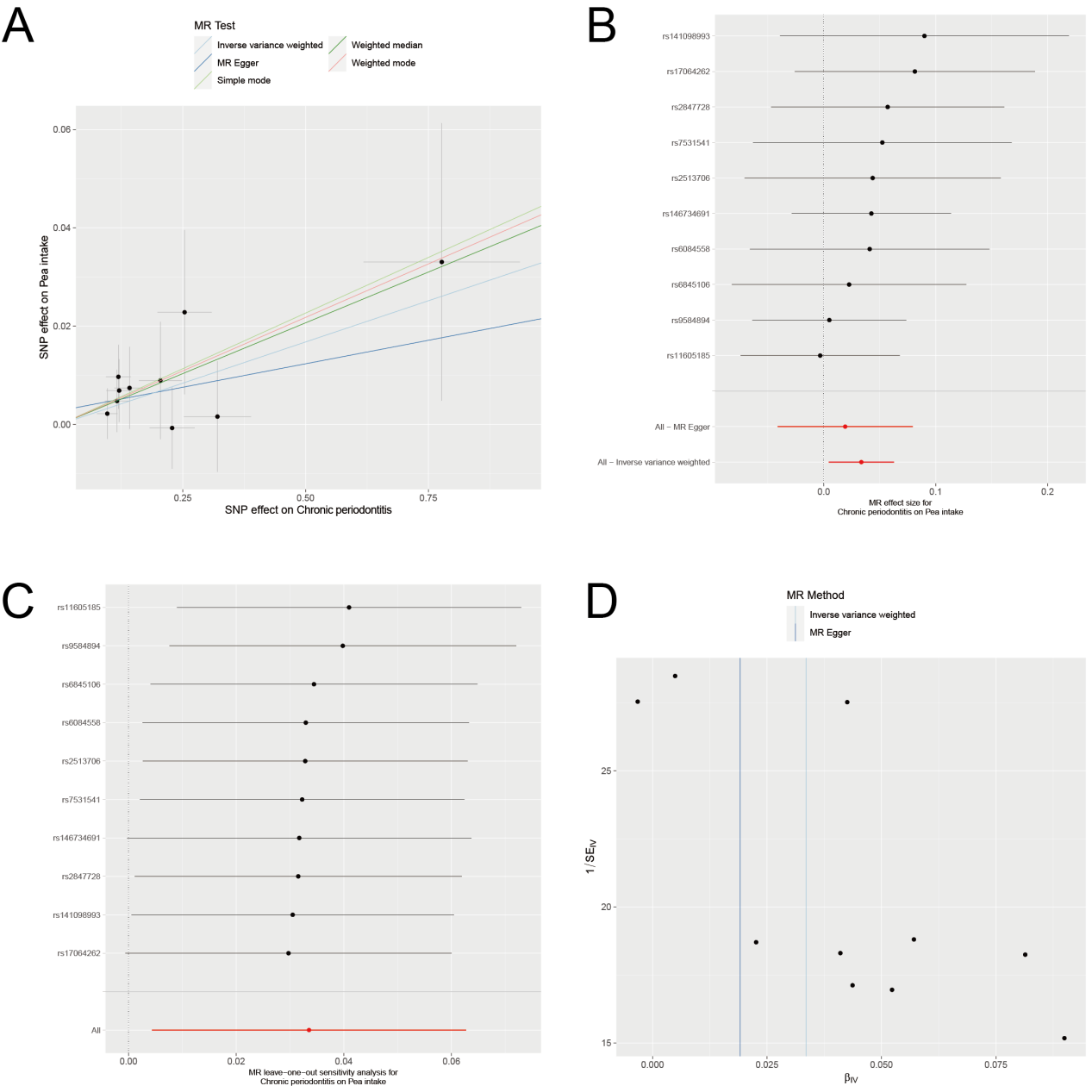


**Supplemental Fig.** **11** **Sensitivity analysis of MR estimates on the association between periodontitis and pea intake. (A)** Scatter Plot: Displays the associations between SNPs and outcomes versus SNPs and exposures, providing effect estimates for individual variants. Different colored lines represent regression slopes fitted by various MR methods. **(B)** Forest Plot: Shows the effect estimate of each SNP, accompanied by 95% confidence intervals. **(C)** Leave-One-Out Analysis: Recalculates effect estimates after sequentially excluding each SNP to determine if a particular SNP significantly influences the association. **(D)** Funnel Plot: Plots estimates against their precision, assessing potential data asymmetry. MR, mendelian randomization; SNP, single-nucleotide polymorphism; β, effect size; SE, standard error.


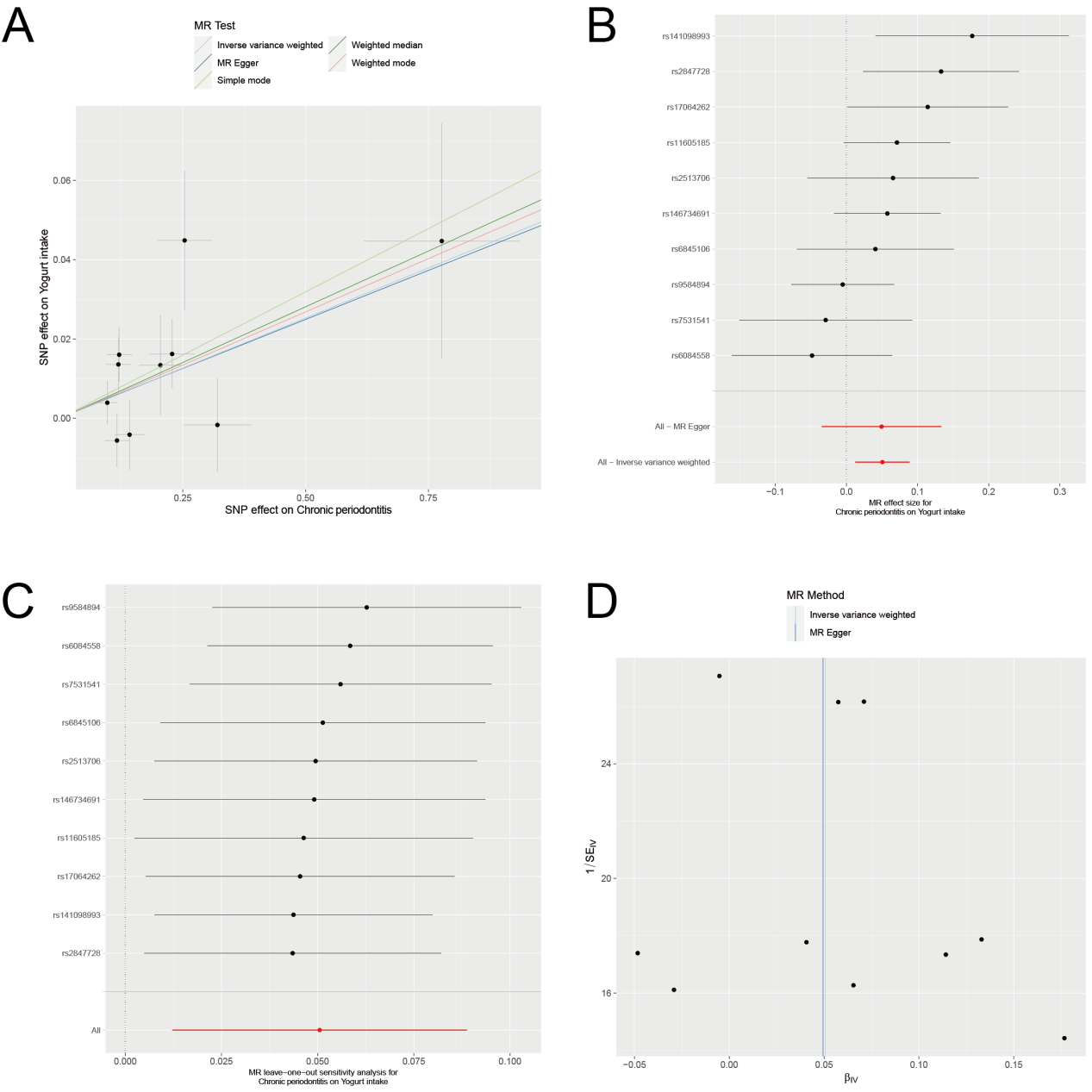


**Supplemental Fig. 12** **Sensitivity analysis of MR estimates on the association between periodontitis and yogurt intake. (A)** Scatter Plot: Displays the associations between SNPs and outcomes versus SNPs and exposures, providing effect estimates for individual variants. Different colored lines represent regression slopes fitted by various MR methods. **(B)** Forest Plot: Shows the effect estimate of each SNP, accompanied by 95% confidence intervals. **(C)** Leave-One-Out Analysis: Recalculates effect estimates after sequentially excluding each SNP to determine if a particular SNP significantly influences the association. **(D)** Funnel Plot: Plots estimates against their precision, assessing potential data asymmetry. MR, mendelian randomization; SNP, single-nucleotide polymorphism; β, effect size; SE, standard error.


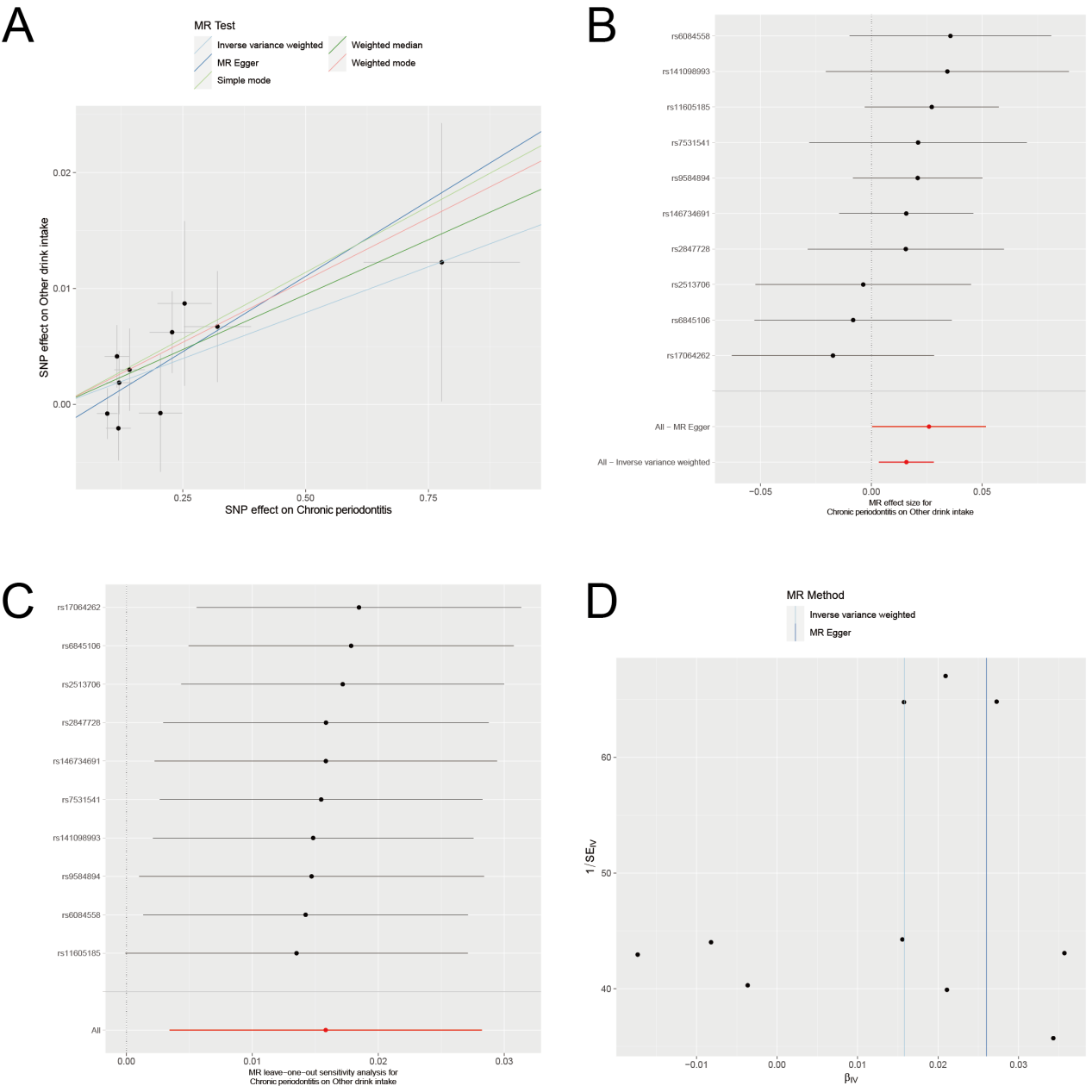


**Supplemental Fig. 13** **Sensitivity analysis of MR estimates on the association between periodontitis and other drink intake. (A)** Scatter Plot: Displays the associations between SNPs and outcomes versus SNPs and exposures, providing effect estimates for individual variants. Different colored lines represent regression slopes fitted by various MR methods. **(B)** Forest Plot: Shows the effect estimate of each SNP, accompanied by 95% confidence intervals. **(C)** Leave-One-Out Analysis: Recalculates effect estimates after sequentially excluding each SNP to determine if a particular SNP significantly influences the association. **(D)** Funnel Plot: Plots estimates against their precision, assessing potential data asymmetry. MR, mendelian randomization; SNP, single-nucleotide polymorphism; β, effect size; SE, standard error.


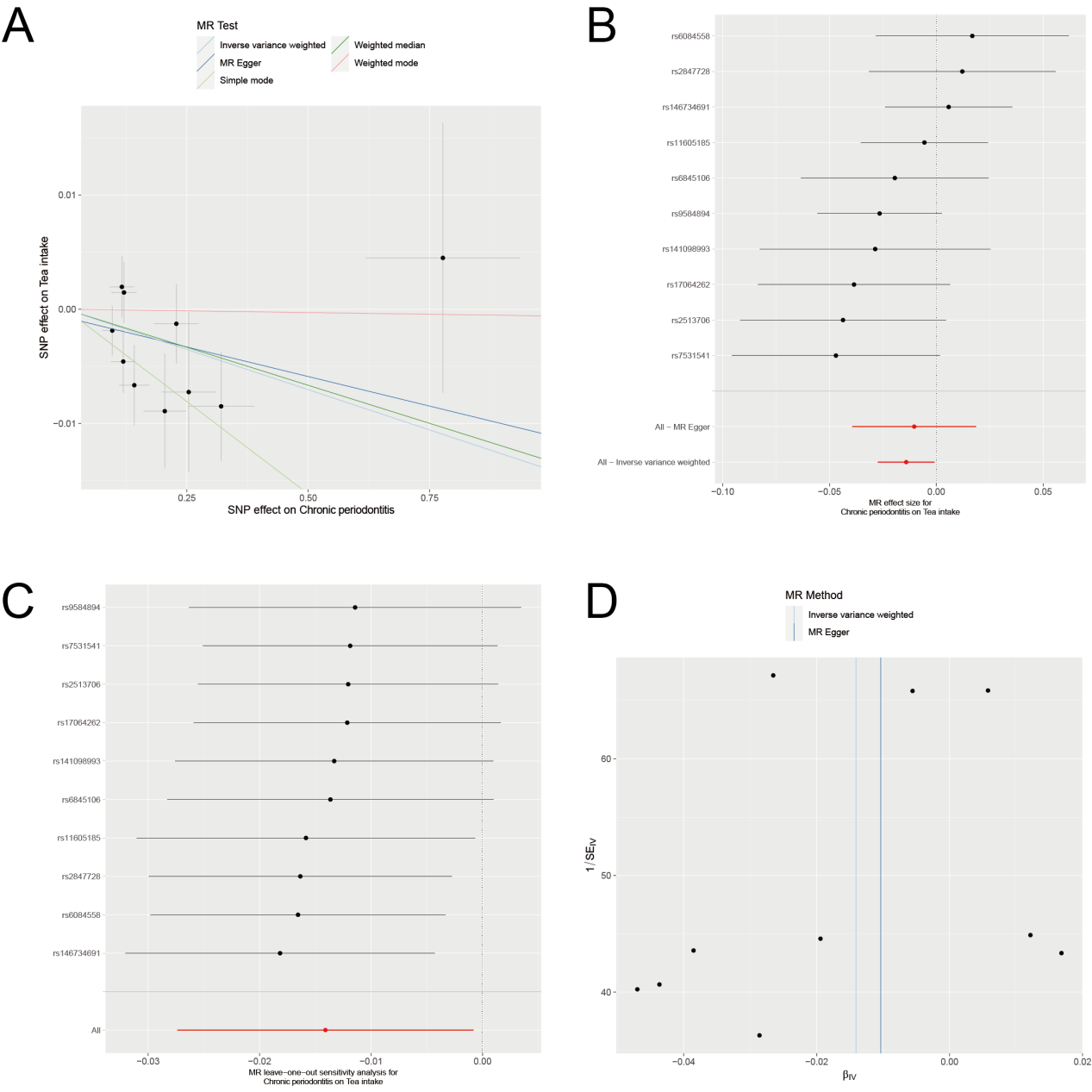


**Supplemental Fig. 14** **Sensitivity analysis of MR estimates on the association between periodontitis and tea intake. (A)** Scatter Plot: Displays the associations between SNPs and outcomes versus SNPs and exposures, providing effect estimates for individual variants. Different colored lines represent regression slopes fitted by various MR methods. **(B)** Forest Plot: Shows the effect estimate of each SNP, accompanied by 95% confidence intervals. **(C)** Leave-One-Out Analysis: Recalculates effect estimates after sequentially excluding each SNP to determine if a particular SNP significantly influences the association. **(D)** Funnel Plot: Plots estimates against their precision, assessing potential data asymmetry. MR, mendelian randomization; SNP, single-nucleotide polymorphism; β, effect size; SE, standard error.
